# A Wavelet Derivative Spectrum Length Method of TFBG Sensor Demodulation

**DOI:** 10.3390/s23042295

**Published:** 2023-02-18

**Authors:** Sławomir Cięszczyk, Krzysztof Skorupski, Martyna Wawrzyk, Patryk Panas

**Affiliations:** 1 Institute of Electronics and Information Technology, Lublin University of Technology, Nadbystrzycka 38A, 20-618 Lublin, Poland; 2 Doctoral School, Lublin University of Technology, Nadbystrzycka 38D/406, 20-618 Lublin, Poland

**Keywords:** TFBG sensors, refractive index, optical spectra analysis

## Abstract

Fibre optic sensors using tilted fibre Bragg grating (TFBG) have high sensitivity for refractive index measurements. In order to achieve good metrological parameters of the measurement, an appropriate method of spectrum demodulation must be used. The method proposed in the article is an improvement of the spectral length algorithm. The spectral length parameter is treated as the sum of the derivative filter responses. In the original version, the first difference of spectrum elements was used, while this article proposes to use the wavelet transform to calculate the numerical derivative approximation. The advantage of this solution is an easy way to select the level of smoothing filtration by changing the scale parameter. The derivation is appropriate even for a relatively low signal-to-noise level. The approximation of the spectral length by the derivative calculated using the wavelet transform eliminates the high-frequency noise of the optical signal. The absolute value of determined spectral derivatives after significant smoothing can be used to estimate the wavelength of the decay of modes. After analyzing experimental data and performing calculations, it turns out that this is a linear method with better resolution than the contour length algorithm.

## 1. Introduction

Tilted fibre Bragg grating (TFBG) has an interesting transmission spectrum that is sensitive to changes in several physical quantities such as temperature [1], bending [2], strain [3], and polarisation state [4]. TFBG gratings can also be used to measure several parameters simultaneously [5,6,7,8]. As with any fibre optic sensor, the main advantage is the small size of the sensor. The most interesting sensing application of such periodic structures is the measurement of the refractive index. The change in the grating spectrum under the influence of the surrounding refractive index (SRI) is significant and thus enables high-resolution measurement. Calibration and subsequent determination of the SRI value based on the transmission spectrum is not a simple task. Intuitive spectrum demodulation methods, such as the most popular envelope method [9], do not use all points of the measured transmission spectrum. It is a large number of measured wavelengths, which is the main advantage of spectrum measurement. It makes it possible to reduce the influence of noise on the determined SRI value and to obtain the algorithmic selectivity of the measurement.

By carrying out a literature review of the TFBG spectrum demodulation methods, a certain systematization of the methods can be made. A single mode can be analyzed for a narrower range of SRI changes [10]. Changes in the entire spectrum can be useful for measuring a wide range of refractive indices [9,11]. Demodulation may consist of indirect determination of the single mode wavelength shift or the so-called cut-off wavelength for the entire spectrum [12]. The amplitude spectral parameters can be the peak amplitude of a single mode or parameters such as the area occupied by all cladding modes. Global amplitude methods also include those that use neural network methods [13].

The biggest problem in global methods for determining cut-off wavelength shifts is the difficulty in ensuring the continuity of this parameter with small changes in the SRI. This difficulty results from the shape of the spectrum consisting of several dozen modes. The basic core mode related to the Bragg wavelength in tilted gratings depends on the effective refractive index of the core *n_eff_*, grating period Λ, but also on the tilt angle *θ* [14]:

(1) $$\lambda_{B}=\frac{2\cdot n_{eff}\cdot \Lambda }{cos\theta }.$$

The wavelength of the cladding resonances can be calculated taking into account the effective refractive index of the core *n_eff,cor_* and the cladding *n_eff,cla_* for the wavelength of a given mode [14]:

(2) $$\lambda_{cla}^{i}=\frac{\left(n_{eff,cor}^{i}+n_{eff,cla}^{i}\right)\cdot \Lambda }{cos\theta }.$$

It is difficult to correctly determine the cut-off wavelength changes from one mode to the next. Intermediate values can only be approximated. This corresponds to relatively large jumps in the calculated SRI value. The method to reduce such jumps may be the averaging of the cut-off wave calculated separately for the lower and upper envelopes of the modes [15]. Reducing the distance between individual modal resonances is possible with double and shifted mode comb grating [16,17].

Techniques used for Surface plasmon resonance (SPR) in TFBG complete the review of spectrum demodulation methods. Spectral shifts of the SPR effect gratings are different than those of the TFBGs themselves. SPR spectrum demodulation methods cannot be directly applied to TFBG spectrum demodulation. However, they can be an inspiration to create new algorithms tailored to a given sensor. Spectrum demodulation methods with the SPR effect, similar to grids without this effect, can be divided into those that use changes in the position of certain spectral features along the wavelength [18] and changes in the transmission amplitude level of the spectrum. A specific range of wavelengths or a single mode can be analyzed. The use of mode amplitude changes as an indicator of SRI can be preceded by an analysis of the sensitivity of a certain range of modes. Such a procedure indicates the most sensitive mode and improves the resolution of the method [19].

From the review of the literature on the demodulation of TFBG spectra, it appears that the quantitative analysis of data measured with optical fibre periodic structures is based on a variety of mathematical techniques. They are aimed at extracting relevant information and improving metrological parameters such as measurement resolution and selectivity. One of the tasks of the developed algorithms is to remove high-frequency noise. Individual methods are developed for a specific type of measurement data. Many of these methods are suitably modified methods known and used in the field of signal processing. The SRI value can be determined based on the frequency distribution shift in the Fourier domain [20]. The optical spectrum can also be modulated by a system with a cavity [21]. For such a system, in the Fourier frequency spectrum, as the SRI increases, the band related to the frequency of the cladding modes decreases and then disappears. The measured signal can be pre-processed using methods such as Fourier transform. In relation to it, the wavelet transform has the advantage of preserving information about the frequency content while simultaneously localizing it in time. In addition, the signal can be represented using different scales. The wavelet transform is used to process spectrophotometric spectra. With its help, the measured signal is decomposed into components of relatively simple shapes, which are subject to scaling and shifting. It appears especially when there is significant noise in the measured spectra [22,23,24]. Its main applications are to replace very popular derivative spectrophotometry methods [25,26].

Calculation of derivatives in the analysis of absorbance spectra is a popular technique for enhancing interesting features, such as the separation of individual peaks from different components [27]. At the same time, the baseline and its fluctuations are compensated. Unfortunately, the determination of the first and subsequent derivatives reduces the signal-to-noise ratio. Real spectra can contain a significant amount of noise. The solution to this drawback is simultaneous filtration along with the determination of derivatives. Unfortunately, it can cause a change in the shape of the spectra and, thus, the loss of information. Differentiating filters should be characterized by the best representation of the spectrum for a specific band in which its signal characteristics are contained. At the same time, above the cut-off frequency, the signal should be attenuated, which corresponds to the reduction in the noise content. With small SNR values, the continuous wavelet transform (CWT) [28] is suitable for determining derivatives. If the spectrum consists of spectral lines with shapes similar to Gaussian ones, then for individual peaks (maximums in the spectrum) for the first derivative, we obtain appropriate zero crossings. In the presence of noise, reducing its level is always associated with a simultaneous reduction in the high-frequency elements of the signal.

In this article, we propose to develop the spectral contour length method using the wavelet transform. We treat the length of the spectrum as a parameter on the basis of which we determine the SRI value [29]. However, for the basic version of this method, the length parameter is calculated as the sum of the elements of the differentiating filter (first difference). The development and improvement of this method consist of determining the derivative of the measured spectrum using the wavelet transform. This leads to noise filtration and improved measurement resolution. Then we use the shape of the spectral derivative to estimate the cut-off mode wavelength. This parameter moves linearly along the wavelength axis with increasing SRI.

## 2. Derivation of Derivatives Using the Wavelet Transform

Wavelet transform of a function *f*(*t*) at scale *s* (dilation parameter) and translation parameter *b* is expressed by the following integral:

(3) $$X_{w}\left(s,b\right)=\frac{1}{\sqrt{s}}\overset{\infty }{\underset{-\infty }{\int }}f\left(t\right)\cdot \psi \left(\frac{t-b}{s}\right)$$

where $\psi \left(t\right)$ is a finite energy continuous function called a mother wavelet. The above integral for a given scale s can be expressed using the convolution operation as [28]:

(4) $$X_{w}\left(s,b\right)=f\left(b\right)⨂\psi^{*}\left(s,b\right).$$

Convolution is an operation that is performed during filtration. If we assume that the function $\psi \left(t\right)$ is the impulse response of the filter, then the result of the transform is the response of such a filter to the input signal. Thus let us write Equation (4) as the filtering equation of the input signal *x*(*b*), transforming it into the output signal *y*(*b*):

(5) $$y\left(b\right)=x\left(b\right)⨂\psi^{*}\left(s,b\right).$$

Equation (5) can be transformed on both sides using the Fourier transform. Taking into account the scaling of the mother wavelet, for a given scale *s*, we write [30]:

(6) $$Y\left(s,\omega \right)=X\left(\omega \right)\cdot \psi \left(-s\cdot \omega \right)\cdot \sqrt{s}.$$

Therefore, the spectrum of the mother function dilated through s will determine the type of filtering in the frequency domain. If the function $\psi \left(t\right)$ was created using the smooth derivative of the function $\Theta \left(t\right)$:

(7) $$\psi \left(t\right)=-\frac{d\theta \left(t\right)}{dt},$$

which has a fast decay property confirmed by the vanishing moment. As a result, the wavelet transform on a given scale can be written as [31]:

(8) $$y\left(t\right)=s\cdot \frac{d}{dt}\left(x\left(t\right)⨂\theta \left(t\right)\right).$$

So, we can write Equation (7) in the domain of the Fourier transform:

(9) $$\psi \left(-s\cdot \omega \right)=\sqrt{s}\cdot \theta \left(-s\cdot \omega \right)\cdot i\omega ,$$

**Figure 1:**
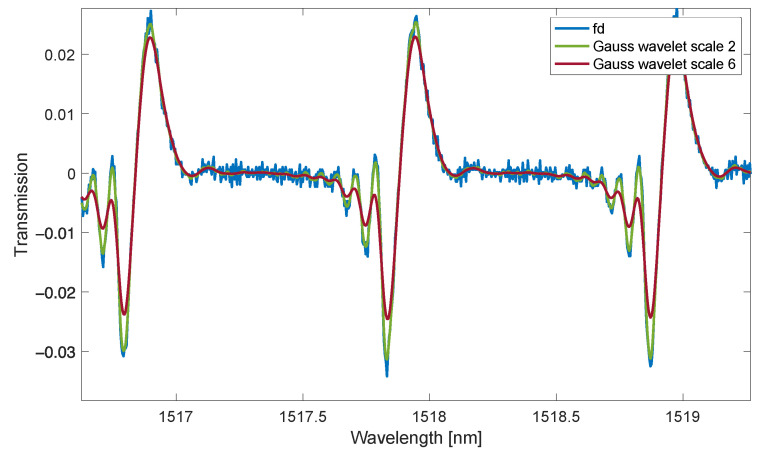
The first derivative of the TFBG grating spectrum is calculated by the first difference method and the wavelet transform method (Gauss wavelet scales 2 and 6).

At the same time, attention should be paid to the shape of the spectrum of the function $\theta \left(-s\cdot \omega \right)$. For an ideal derivative filter, the spectrum of this function should be a constant component. The condition for differentiation with the use of the wavelet transform is that the function $\theta \left(t\right)$ has the character of the impulse response of the low-pass filter. Differentiation will, therefore, only take place within a certain and limited frequency range. The wavelet transforms for a given scale is a low-pass differentiator filter. The dilation parameter changes the cut-off frequency of low-band filtration and, thus, the range of frequencies for which differentiation occurs. The properties of the derivative approximation using CWT depend mainly on the analysis wavelet used. The greater blurring of shapes occurs when the scale parameter is increased (Figure 1).

**Figure 2:**
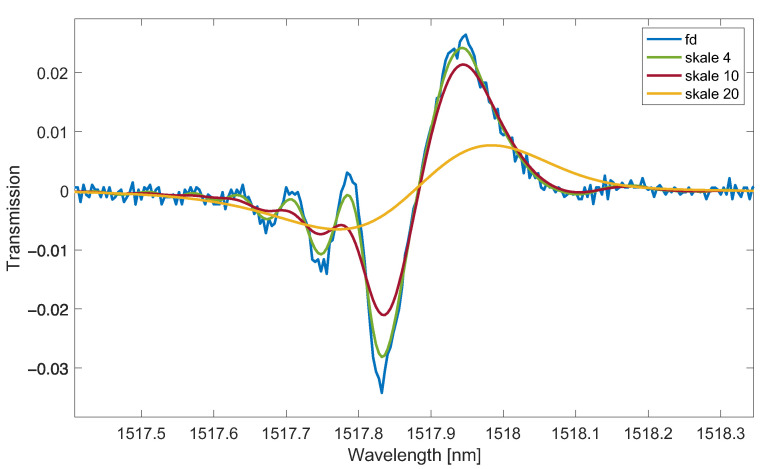
Change of the spectrum of the first derivative for larger values of the scale parameter (Gauss wavelet, scale 4, 10, 20).

The improvement of the signal-to-noise ratio is obtained by increasing the scale parameter, which causes stronger low-band filtering. For a smaller scale parameter, the wavelet transform has a better resolution in the fundamental domain (typically time) and worse in the frequency domain. The scale-dependent expression $\psi \left(-s\cdot \omega \right)$ is the frequency response of the filter from equation 4. Increasing the scale means narrowing the frequency spectrum of the wavelet filter. This also corresponds to a reduction in the resolution of the processed transmission spectra. The optimal selection of the dilation coefficient, therefore, depends on the shape of the signal and the signal-to-noise ratio. It is often based on an analysis of the shape of the obtained derivatives. Comparing the wavelet numerical derivative with the derivative calculated analytically for test signals for small-scale factors, the resolution of the spectra (peak widths) is similar. An increase in the dilation scale results in greater smoothing and reduction in the processed peaks in the optical signal (Figure 2).

## 3. Derivatives and Contour Length for TFBG Spectra

**Figure 3:**
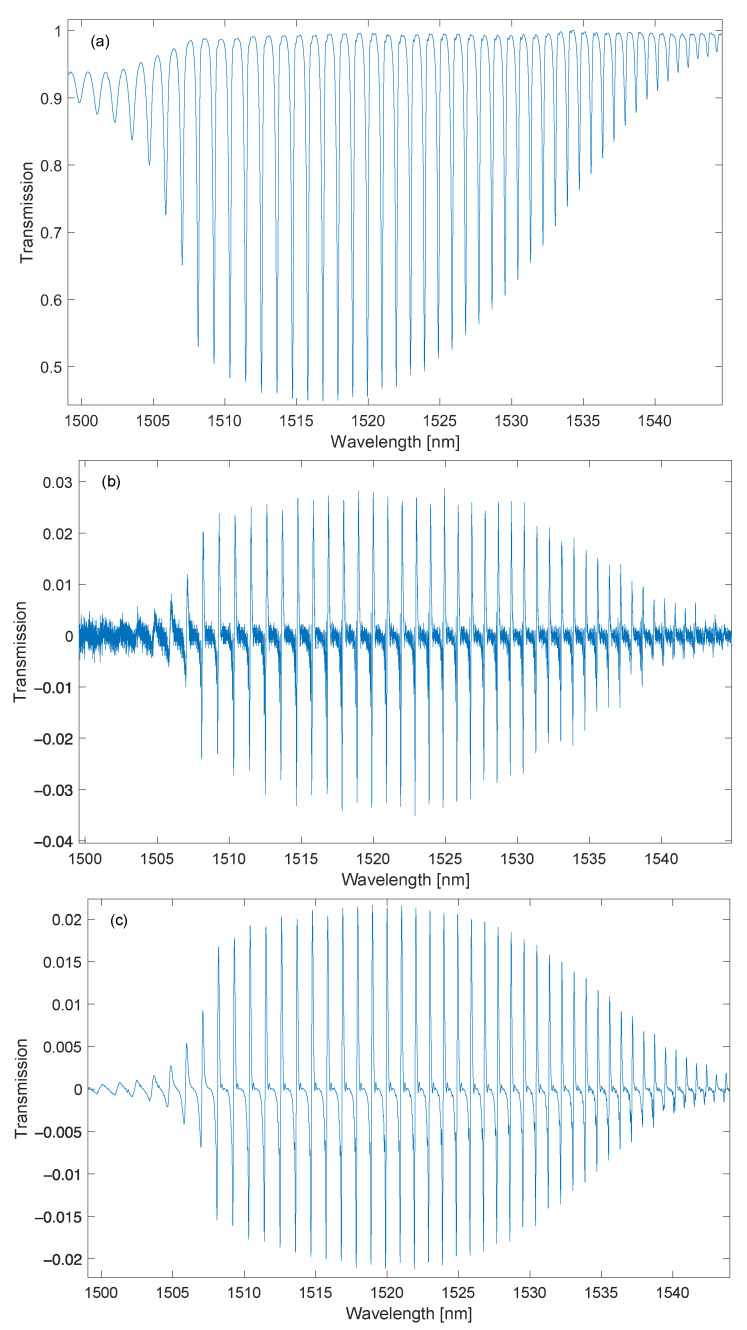
The global spectrum of cladding modes (**a**) and its derivative are calculated by the first difference method (**b**) and the wavelet transform for a scale of 10 (**c**).

The TFBG spectrum consists of dozens of cladding modes. This spectrum depends on the tilt angle of the refractive index changes. Figure 3 shows an example of the measured spectrum of the TFBG grating with 6 degrees tilt. We carried out the measurement without averaging (repeating) the spectra in the spectrum analyzer. As a result, the measured spectrum has a relatively high noise content. The basis of the demodulation algorithm, referred to as the length of the spectrum contour or the length of the spectrum, is the determination of the sum of the differences between successive values of the spectrum amplitudes. This corresponds to the sum of the first derivative of the function. This algorithm was proposed for the analysis of spectra of FTIR spectrometers [32,33]. The differential equation of a suitable digital filter with a finite impulse response length can be written as follows:

(10) $$y\left[n\right]=x\left[n\right]-x\left[n-1\right].$$

where $x\left[n\right]$ is the input signal of the filter, which in the considered aspect is the measured transmission spectrum. The shape of the output signal $y\left[n\right]$ of the filter for an example spectrum is shown in Figure 3b. A characteristic feature of derivatives is the amplification of high-frequency noise. This phenomenon is very well visible in the spectrum in Figure 3b. In the range of 1500–1505 nm, the noise is even more visible than the derivatives of individual transmission peaks. In the further part of the spectrum, the noise is visible against the background of the proper signal with larger amplitudes. The derivative can also be computed using the wavelet transform. An example spectrum calculated using continuous wavelet transform, Gauss wavelet, and scale 10 is shown in Figure 3c. Comparing the spectra from Figure 3b,c, a significant reduction in noise for calculations made using the wavelet transform is noticeable.

The length of the spectrum is a parameter indicating the changes in the spectrum associated with the leak of individual cladding modes. The decrease in the spectral length value is correlated with the increase in the SRI value. We calculate the length of the spectrum by summing up the absolute value of the derivative filter response:

(11) $$CL=\overset{N-1}{\underset{i=0}{\sum }}\left|x\left[n\right]-x\left[n-1\right]\right|=\overset{N-1}{\underset{i=0}{\sum }}\left|y\left[n\right]\right|.$$

If the spectrum of the first derivative calculated using the wavelet transform (wavelet filter) is marked as $y_{w}\left[n\right]$ the length of the spectrum will then be:

(12) $$CL_{w}=\overset{N-1}{\underset{i=0}{\sum }}\left|y_{w}\left[n\right]\right|.$$

**Figure 4:**
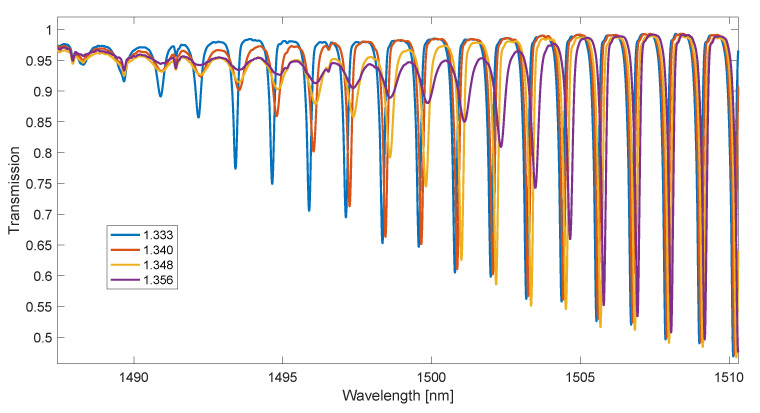
Part of the spectrum is used to calculate the contour length, for example, four SRI values.

In the first experiment, 19 glucose concentrations with distilled water were measured. SRI values ranged from 1.333 to 1.3606. A 6° tilt grating was fabricated by the phase mask method using an excimer laser (Coherent Inc., Santa Clara, CA, USA). Before the recording process, the single-mode optical fibre was placed in a hydrogen chamber in order to increase its sensitivity to laser radiation. During the refractive index measurements, a super-luminescent diode (SLED) S5FC1005S (Thorlabs Inc., Newton, NJ, USA) was used as the radiation source. The transmission spectrum of the gratings would be measured with an AQ6370D optical spectrum analyzer (Yokogawa, Tokyo, Japan) with a resolution of 0.004 nm. For each SRI value, 20 spectral measurements were made. Example spectra are shown in Figure 4.

## 4. Comparison of First Difference and Wavelet Transform for Calculation of Derivative and Spectrum Length

**Figure 5:**
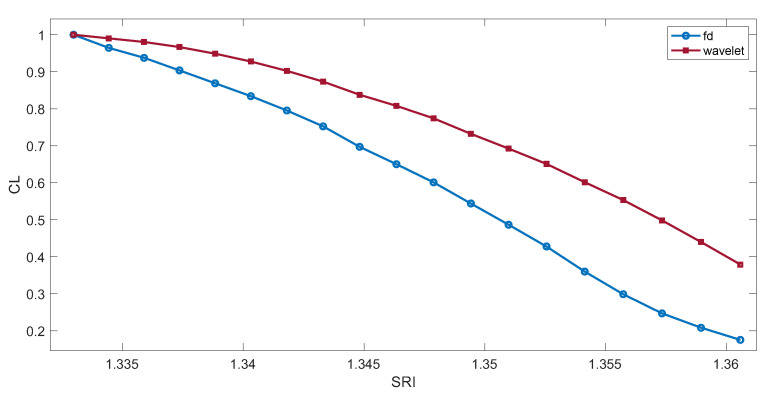
The length of the spectrum contour depends on the SRI value. Calculations by the first difference method and using the wavelet transform for the scale of 40.

Figure 5 shows the characteristics of the dependence of the normalized contour length depending on the SRI. The range of the spectrum for which the contour length was calculated is shown in Figure 4. Increasing the scale of the wavelet filter causes it to become a derivative filter with an additional low-pass filtering effect. The differentiated spectrum becomes smoother and has smaller amplitudes. The length of the spectral contour for a larger scale will have smaller values. If we normalize this length to unity, then for the same range of wavelengths, its value for the maximum measured SRI will have a greater value than in the case of calculation using the first difference method (Figure 5). In the course of the research, both continuous and discrete wavelet transforms were analyzed. Different types of wavelets were also tested [34]. The Gauss-type wavelet for the continuous wavelet derivative was used for the calculations. Calculations of the spectrum derivative were performed on a scale from 2 to 100. The best resolution was obtained on a scale of 40.

**Figure 6:**
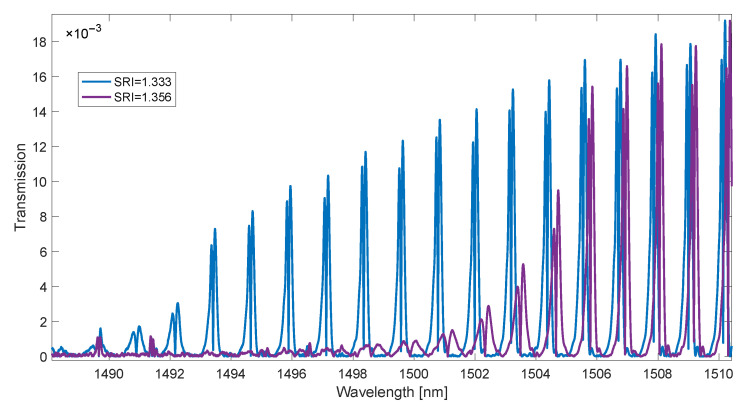
Absolute value of the first spectral difference was measured for two SRI values.

**Figure 7:**
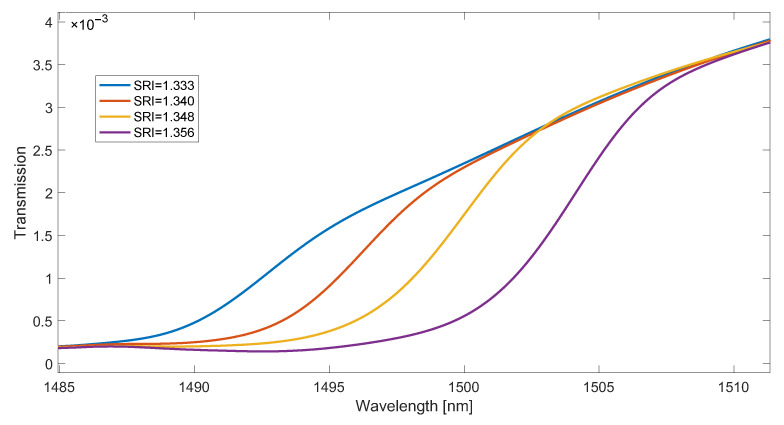
Smoothed shape of the rectified derivative of the spectrum for four SRI values, including two in Figure 6.

**Figure 8:**
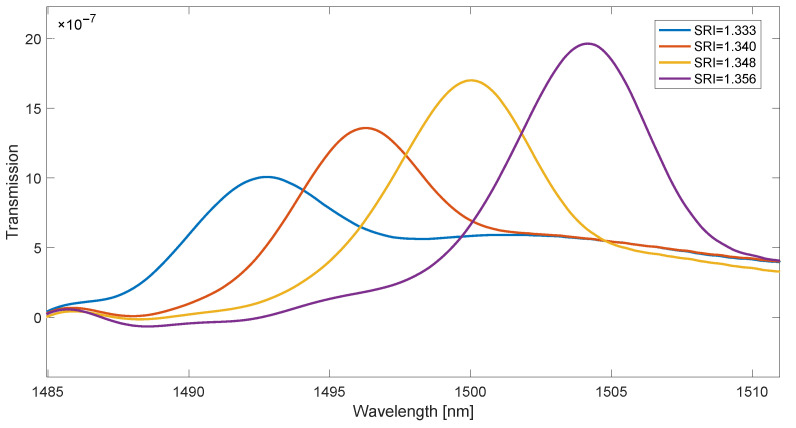
Derivative of the smoothed first derivative absolute value of the spectrum in Figure 7.

The leak of the cladding modes and their envelope shifting towards longer wavelengths with the increase in the SRI value can be used as a method of spectrum demodulation. The shifting of the cut-off wavelength and the leak of successive modes is linear with increasing SRI. This property makes the methods of SRI determination that use an indirect calculation of the cut-off wavelength to be methods with good linearity. The simplest of these methods is to determine the maximum of the first derivative of the envelope of cladding modes. In the case of a spectral contour, the cumulative version can be used for this as a wavelength-dependent function. Another option is to calculate the derivative from the absolute value of the spectrum derivative shown in Figure 6. Since it is a quantity with slow changes with respect to the wavelength, the function of the contour absolute value should be strongly smoothed before determining the derivatives (Figure 7). For this purpose, a non-causal Gaussian moving average filter was used. A properly smoothed curve is obtained for the length of the impulse response of the filters above 1200. The derivative of the smooth curve in Figure 7 is shown in Figure 8.

**Figure 9:**
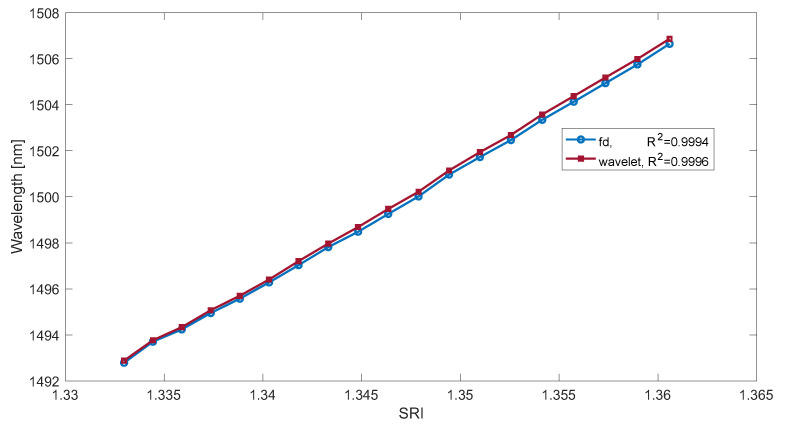
The wavelength of the smoothed derivative maximum of the TFBG absolute contour spectrum.

Figure 9 shows the dependence of the wavelength of the maximum derivative of the smoothed absolute value of the first difference of the TFBG spectrum on the SRI coefficient. Table 1 presents a comparison of the resolution for the four methods considered. In both types of methods, derivatives calculated using the wavelet transform give better results.

**Figure 10:**
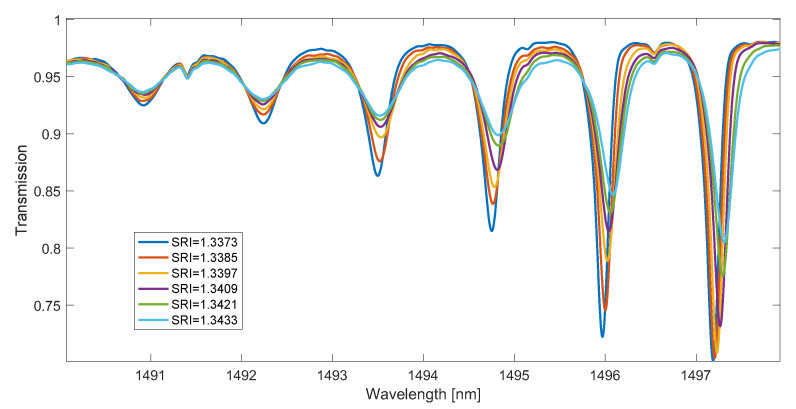
TFBG transmission spectra for small SRI changes.

The main disadvantage of the methods determining the wavelength of the mode leak shifting with the SRI is the presence of steps depending on this wavelength from the SRI. Smoothing the spectrum derivative with the use of a long impulse response FIR filter seems to eliminate this effect. However, in order to verify this, glucose solutions with very small differences in SRI values were measured. In the second experiment, 21 glucose concentrations with distilled water were measured. SRI values ranged from 1.374 to 1.3433. The TFBG spectrum was measured for each single SRI value twenty times. Parts of example spectra in which the greatest changes occur under the influence of SRI in this range are shown in Figure 10. Since the resolution of the spectrometer was 0.004 nm, the number of data points between the spectral peaks is about 320. This value decreases with the wavelength.

**Figure 11:**
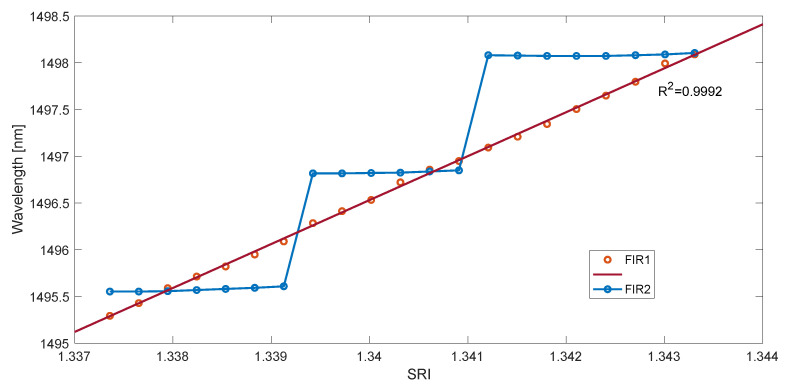
The wavelength of the maximum derivative value of the contour spectrum absolute value is calculated with the first difference method for two lengths of smoothing filters.

**Figure 12:**
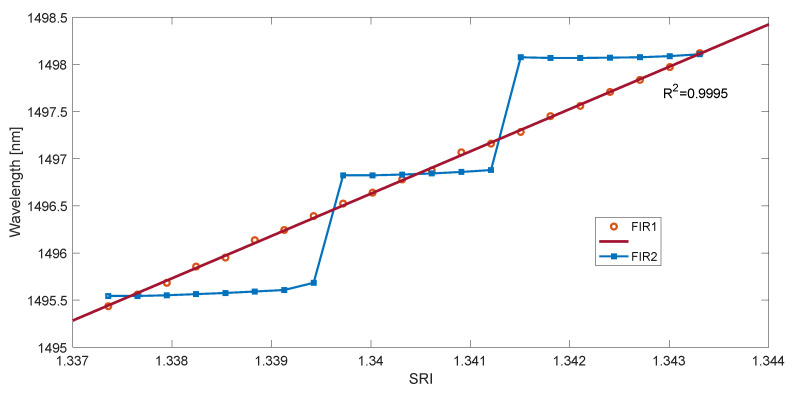
The wavelength of the maximum derivative value of the contour spectrum absolute value was calculated with the wavelet method for two lengths of smoothing filters.

**Figure 13:**
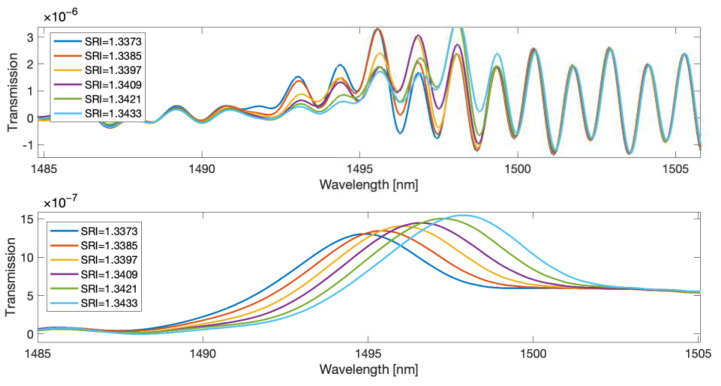
The derivative of the smoothed absolute value of the first difference of the TFBG spectrum for two lengths of the smoothing filter.

Similarly to the first set of experimental data, calculations were carried out for the four considered methods. The measurement characteristics of the determined cut-off wavelength as a function of SRI are shown in Figure 11 and Figure 12. They show the characteristics for the maximum derivative value of the rectified and smoothed contour length spectrum in two cases. They differ in the length of the impulse response of the smoothing filter. If the impulse response is too short, there are steps in the characteristic. This is due to the jump of the maximum of the second derivative from the first to the next mode. This effect can be explained by analyzing Figure 13a. If we extend the length of the filter, the value of the first derivative of the spectrum becomes smooth without steps, as shown in Figure 13b. The impulse response length of the filter for Figure 13a was 500, while for Figure 13b, it was 1500.

Taking the derivative of a smooth function also yields a smooth function. The wavelength of the maximum of such a function moves linearly with increasing SRI. The stepped characteristics of Figure 11 and Figure 12 would mean an SRI resolution of 0.002 (2 × 10^−3^), while a sufficiently long filter allows a resolution of 10^−5^ (Table 2).

## 5. Conclusions

The method of contour (spectra) length has been proposed to solve problems in the measurement of spectra with FTIR spectrometers. It also proved effective in demodulating the TFBG spectra. It has become a popular method in the scientific community dealing with TFBGs, as evidenced by the number of citations in the first article. In the basic version, this is the numerically simplest method because the sum of the absolute values of the first signal (spectrum) differences is calculated. The first difference is the approximation of the spectrum derivative. Hence, the contour length method can be extended to the calculation of the first derivative by other methods. This article proposes an algorithm for calculating the approximation of spectral derivatives using the wavelet transform. We have shown that this method works well in the case of noise in the measured spectra. By selecting the appropriate scale of the wavelet, the resolution of the algorithm was improved compared to the first difference method. In addition, it was shown that the method of the maximum derivative of the smoothed (filtered) absolute derivative of the transmission spectrum is characterized by good linearity. A noteworthy achievement during the work on this article is the demonstration of the linearity of the cut-off wavelength determination characteristics, even with very small changes in SRI. This property is ensured by a properly selected smoothing filter.

## Figures and Tables

**Table 1:** Comparison of the resolution for the contour length methods and the maximum derivative of the smoothed local spectral contour value in the case of the derivative calculated by the first difference method and the wavelet transform method.

Method	Resolution
Contour length, first difference	2.3 × 10^−5^
Contour length, wavelet difference	1.2 × 10^−5^
Maximum of derivative, first difference	2.1 × 10^−5^
Maximum of derivative, wavelet difference	1.75 × 10^−5^

**Table 2:** Comparison of the resolution for the contour length methods and the maximum derivative value of the contour in the case of the derivative calculated by the first difference method and the wavelet transform method.

Method	Resolution
Contour length, first difference	1.95 × 10^−5^
Contour length, wavelet difference	1.04 × 10^−5^
Maximum of derivative, first difference	0.83 × 10^−5^
Maximum of derivative, wavelet difference	0.75 × 10^−5^

## Data Availability

Not applicable.

## References

[1] Zhao J., Wang H., Sun X. (2021). Study on the performance of polarization maintaining fiber temperature sensor based on tilted fiber grating. Measurement.

[2] Jin Y.X., Chan C.C., Dong X.Y., Zhang Y.F. (2009). Temperature-independent bending sensor with tilted fiber Bragg grating interacting with multimode fiber. Opt. Commun..

[3] Miao Y., Liu B., Zhang W., Dong B., Zhou H., Zhao Q. (2008). Dynamic temperature compensating interrogation technique for strain sensors with tilted fiber Bragg gratings. IEEE Photonics Technol. Lett..

[4] Lu Y., Shen C., Chen D., Chu J., Wang Q., Dong X. (2014). Highly sensitive twist sensor based on tilted fiber Bragg grating of polarization-dependent properties. Opt. Fiber Technol..

[5] Takeda S.I., Sato M., Ogasawara T. (2022). Simultaneous measurement of strain and temperature using a tilted fiber Bragg grating. Sens. Actuators A Phys..

[6] Alberto N.J., Marques C.A., Pinto J.L., Nogueira R.N. (2010). Three-parameter optical fiber sensor based on a tilted fiber Bragg grating. Appl. Opt..

[7] Fazzi L., Struzziero G., Dransfeld C., Groves R.M. (2022). A single three-parameter tilted fibre Bragg grating sensor to monitor the thermosetting composite curing process. Adv. Manuf. Polym. Compos. Sci..

[8] Fazzi L., Dias N., Holynska M., Tighe A., Rampini R., Groves R.M. (2022). Monitoring of silicone adhesive in space solar cells with an embedded multi-parameter TFBG sensor in a simulated space environment. Meas. Sci. Technol..

[9] Laffont G., Ferdinand P. (2001). Tilted short-period fibre-Bragg-grating-induced coupling to cladding modes for accurate refractometry. Meas. Sci. Technol..

[10] Miao Y., Liu B., Tian S., Zhao Q. (2009). Temperature-insensitive refractive index sensor based on tilted fiber Bragg grating. Microw. Opt. Technol. Lett..

[11] Fazzi L., Groves R.M. (2020). Demodulation of a tilted fibre Bragg grating transmission signal using őĪ-shape modified Delaunay triangulation. Measurement.

[12] Pham X., Si J., Chen T., Qin F., Hou X. (2019). Wide range refractive index measurement based on off-axis tilted fibre Bragg gratings fabricated using femtosecond laser. J. Light. Technol..

[13] Cao Z., Xia T., Zhang S., Zhang S., Mei Y., Liu Z., Li Z. (2022). Improved Spectral Interrogation of Tilted Fiber Bragg Grating Refractometer Using Residual Convolutional Neural Networks. J. Light. Technol..

[14] Erdogan T., Sipe J.E. (1996). Tilted fiber phase gratings. J. Opt. Soc. Am. A.

[15] Huang Z., Yang N., Xie J., Han X., Yan X., You D., Xiao G. (2022). Improving accuracy and sensitivity of a tilted fiber Bragg grating refractometer using cladding mode envelope derivative. J. Light. Technol..

[16] Skorupski K., Cięszczyk S., Panas P. (2021). The Structure and Preparation Method of Spectrally Shifted Double-Comb Tilted Fibre Bragg Gratings. IEEE Photonics Technol. Lett..

[17] Cięszczyk S., Skorupski K., Panas P. (2022). Single-and Double-Comb Tilted Fibre Bragg Grating Refractive Index Demodulation Methods with Fourier Transform Pre-Processing. Sensors.

[18] Manuylovich E., Tomyshev K., Butov O.V. (2019). Method for determining the plasmon resonance wavelength in fiber sensors based on tilted fiber Bragg gratings. Sensors.

[19] Lin W., Huang W., Liu Y., Chen X., Qu H., Hu X. (2022). Cladding Mode Fitting-Assisted Automatic Refractive Index Demodulation Optical Fiber Sensor Probe Based on Tilted Fiber Bragg Grating and SPR. Sensors.

[20] Udos W., Lim K.S., Tan C.L., Ismail M.N., Ooi C.W., Zakaria R., Ahmad H. (2020). Spatial frequency spectrum of SPR-TFBG: A simple spectral analysis for in-situ refractometry. Optik.

[21] Paladino D., Quero G., Caucheteur C., Mégret P., Cusano A. (2010). Hybrid fiber grating cavity for multi-parametric sensing. Opt. Express.

[22] Xi Y., Li Y., Duan Z., Lu Y. (2018). A novel pre-processing algorithm based on the wavelet transform for Raman spectrum. Appl. Spectrosc..

[23] Wahab M.F., O’Haver T.C. (2020). Wavelet transforms in separation science for denoising and peak overlap detection. J. Sep. Sci..

[24] Zhang F., Liu J., Lin J., Wang Z. (2019). Detection of oil yield from oil shale based on near-infrared spectroscopy combined with wavelet transform and least squares support vector machines. Infrared Phys. Technol..

[25] Hassan S.A., Abdel-Gawad S.A. (2018). Application of wavelet and Fuorier transforms as powerful alternatives for derivative spectrophotometry in analysis of binary mixtures: A comparative study. Spectrochim. Acta Part A Mol. Biomol. Spectrosc..

[26] Elzanfaly E.S., Hassan S.A., Salem M.Y., El-Zeany B.A. (2015). Continuous Wavelet Transform, a powerful alternative to Derivative Spectrophotometry in analysis of binary and ternary mixtures: A comparative study. Spectrochim. Acta Part A Mol. Biomol. Spectrosc..

[27] Shao X., Cui X., Wang M., Cai W. (2019). High order derivative to investigate the complexity of the near infrared spectra of aqueous solutions. Spectrochim. Acta Part A Mol. Biomol. Spectrosc..

[28] Nie L., Wu S., Lin X., Zheng L., Rui L. (2002). Approximate derivative calculated by using continuous wavelet transform. J. Chem. Inf. Comput. Sci..

[29] Cięszczyk S., Harasim D., Kisała P. (2017). A novel simple TFBG spectrum demodulation method for RI quantification. IEEE Photon. Tech. Lett..

[30] Messina A. (2004). Detecting damage in beams through digital differentiator filters and continuous wavelet transforms. J. Sound Vib..

[31] Zhang X., Jin J. (2004). Wavelet derivative: Application in multicomponent analysis of electrochemical signals. Electroanal. Int. J. Devoted Fundam. Pract. Asp. Electroanal..

[32] Bak J. (2001). Retrieving CO concentrations from FT-IR spectra with nonmodeled interferences and fluctuating baselines using PCR model parameters. Appl. Spectrosc..

[33] Kozlov D., Besov A. (2011). Method of spectral subtraction of gas-phase fourier transform infrared (FT-IR) spectra by minimizing the spectrum length. Appl. Spectrosc..

[34] Luo J., Bai J., Shao J. (2006). Application of the wavelet transforms on axial strain calculation in ultrasound elastography. Prog. Nat. Sci..

